# How to enhance your own development as a teacher and learner

**Published:** 2018-07-31

**Authors:** Sally Parsley

**Affiliations:** 1 E-learning Manager: International Centre for Eye Health, London School of Hygiene and Tropical Medicine, London, UK.

**Digital technology can transform the way we learn and teach. Are you ready?**

**Figure:**
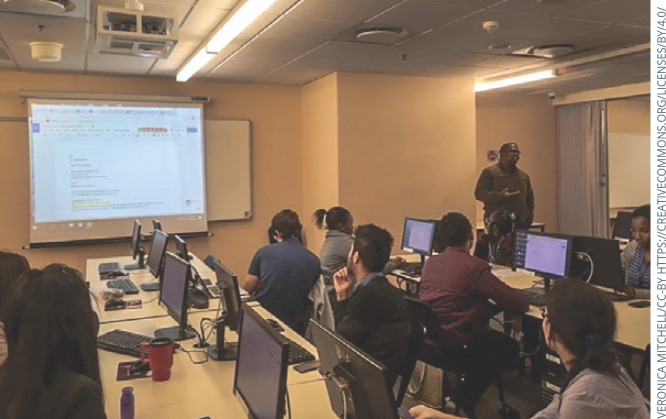
Technology enhanced teaching and learning at the University of Cape Town. SOUTH AFRICA

Digital capabilities are the skills and competencies we need in order to use devices, such as mobile phones, laptops and tablets, that help us to achieve our goals – whether related to work, everyday life, or teaching and learning (Figure 1).

Being digitally capable is an ongoing process of development and change over time and across different contexts. For example, we may be competent at communicating with our friends on our mobile phone but less capable when participating in an e-learning course. Developing our digital capabilities can help us to save time and be more effective as teachers and learners. It can also help us to prepare for the future in a fast-changing technological world.

**Figure 1:**
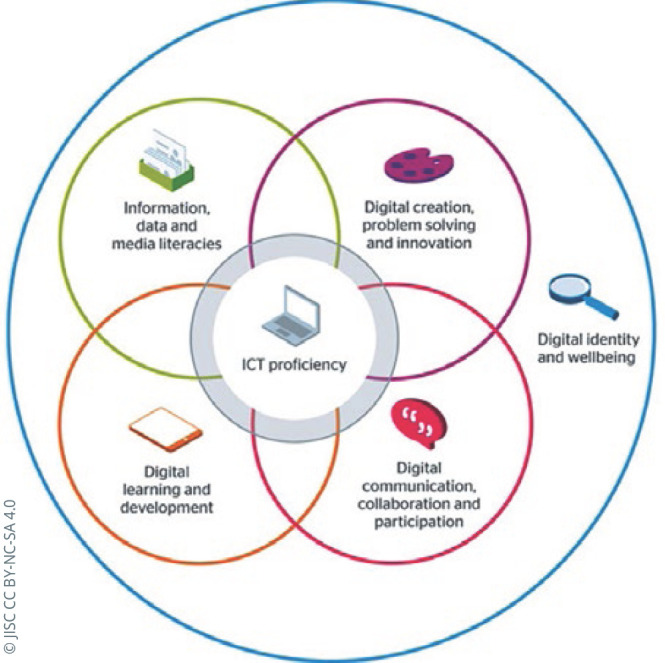
Digital capabilities framework developed for Higher Education in the UK^1^

## Useful resources

Whether you're a student, health worker or healthcare educator, we hope the links below can help you develop your digital capabilities and achieve your teaching and learning goals. Which links look the most important or interesting to you? We suggest you start there first.

### International Council of Ophthalmology Center for Ophthalmic Educators

Courses, curricula, resources and community activities supporting professional development of ophthalmic educators. **https://educators.icoph.org**

### Teaching in online and blended environments

Written for faculty at the University of Cape Town, South Africa, this guide provides an overview of different digitally enhanced blended teaching and learning possibilities. **https://docs.google.com/document/d/17gQ1c1jjiau0DSbZyLD842jc8VpogT9D_QwKotv8gt8/edit**

### ORBIS Cybersight: Ophthalmic Educators' Resources

Curricula and competencies to support blended teaching using Cybersight free online courses: **https://cybersight.org/portfolio/ophthalmic-educators-resources/**

### International Centre for Eye Health & University of Cape Town: Webinars series on technology enhanced teaching and learning for health professionals

3 webinars exploring how health professionals around the world are using digital technologies to prepare for practice, keep their knowledge and skills updated and train others. **http://iceh.lshtm.ac.uk/oer/tel-webinars/**

### Open University (UK) Get started with online learning

Free short course (6 hours) introducing online learning, the skills you need to take part and how to evaluate your own study skillset. **http://www.open.edu/openlearn/education/get-started-online-learning/content-section-overview**

### Accessing good health information and resources

This CEHJ article from last year aims to help eye health workers find and manage digital information for professional development. **https://www.cehjournal.org/article/accessing-good-health-information-and-resources/**

*What is available locally? Is a college, university or professional body in your area offering courses or community activities in information and communications technology (ICTs) or digital skills.*

**Help us find more resouces**

If you know of other good resources for online learning and teaching, we would like to hear from you. Please email **editor@cehjournal.org**
